# Mass Spectrometric Identification of Antimicrobial Peptides from Medicinal Seeds

**DOI:** 10.3390/molecules26237304

**Published:** 2021-12-01

**Authors:** Tessa B. Moyer, Amanda M. Brechbill, Leslie M. Hicks

**Affiliations:** Department of Chemistry, University of North Carolina at Chapel Hill, 125 South Road, Chapel Hill, NC 27599, USA; tbartgesmoyer@gmail.com (T.B.M.); ambrech@email.unc.edu (A.M.B.)

**Keywords:** *Linum* *usitatissimum*, flax, *Trifolium* *pratense*, red clover, *Sesamum* *indicum*, sesame, cysteine-rich, antimicrobial peptides

## Abstract

Traditional medicinal plants contain a variety of bioactive natural products including cysteine-rich (Cys-rich) antimicrobial peptides (AMPs). Cys-rich AMPs are often crosslinked by multiple disulfide bonds which increase their resistance to chemical and enzymatic degradation. However, this class of molecules is relatively underexplored. Herein, in silico analysis predicted 80–100 Cys-rich AMPs per species from three edible traditional medicinal plants: *Linum usitatissimum* (flax), *Trifolium pratense* (red clover), and *Sesamum indicum* (sesame). Bottom-up proteomic analysis of seed peptide extracts revealed direct evidence for the translation of 3–10 Cys-rich AMPs per species, including lipid transfer proteins, defensins, α-hairpinins, and snakins. Negative activity revealed by antibacterial screening highlights the importance of employing a multi-pronged approach for AMP discovery. Further, this study demonstrates that flax, red clover, and sesame are promising sources for further AMP discovery and characterization.

## 1. Introduction

Plants are sessile organisms unable to flee from abiotic and biotic stresses and must produce a diverse array of defensive compounds [1]. Traditional medicines often leverage the activities of these natural products to treat a wide range of diseases [2]. While small-molecule natural products are generally better characterized than their peptide counterparts, investigation of medicinal plant extracts through the analytical lens for larger peptide-like biomolecules can reveal novel antimicrobial peptides (AMPs).

**Figure 1:**
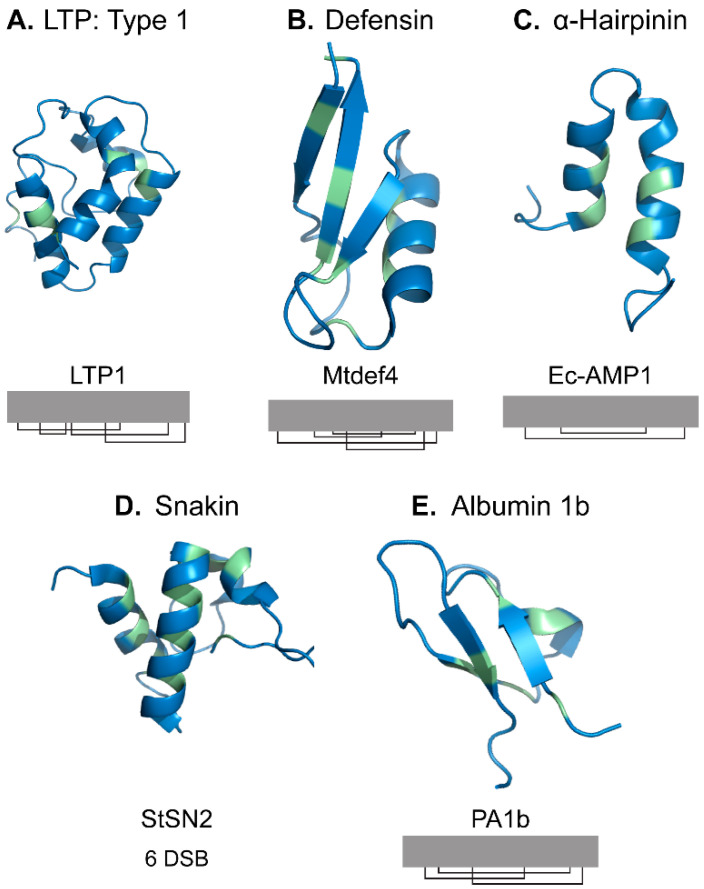
Plant cysteine-rich peptide families are often defined by conserved structural features including disulfide bonds and peptide folding. (**A**) Shown are 9–10 kDa lipid transfer proteins, such as LTP1 (*Oryza sativa*, PDB: 1BV2) [4], that are considered Type 1 and contain four disulfide bonds (I–VI, II–III, IV–VII). (**B**) Defensins, such as MtDef4 (*Medicago truncatula*, 2LR3) [5] usually contain four disulfide bonds (I–VIII, II–V, III–VI, and IV–VII). (**C**) α-Hairpinins, such as EcAMP1 (*Echinochloa crus-galli*, PDB: 2L2R) [6] include two disulfide bonds (I–IV and II–III). (**D**) Snakins contain six disulfide bonds. Their connectivity has only been experimentally determined for *Solanum tuberosum* snakin-1 (PDB: 5E5Q) [7] but in silico modeling suggests that other connectivity is possible [8]. (**E**) AMPs derived from the plant albumin 1 subunit 1b, such as PA1b (*Pisum sativum*, PDB: 1p8b) [9] form three disulfide bonds (I–IV, II–V, III–VI). Figures generated in Pymol. Cysteine residues are colored green.

Endogenous AMPs are usually small (<10 kDa), cationic, and enriched in cysteine (Cys) residues [3]. They are organized into families (e.g., lipid transfer proteins, defensins, α-hairpinins, snakins, and albumin 1b peptides) according to sequence and structural similarities (Figure 1) [3]. Cysteine-rich (Cys-rich) AMPs are often stabilized by multiple disulfide bonds conferring enhanced stability against proteases, temperature, and pH [3]. The enhanced stability of Cys-rich AMPs relative to linear bioactive peptides makes them intriguing components of medicinal botanical extracts.

Historically, plant Cys-rich AMP research has been driven by bioassay-guided discovery. However, the increasing availability of requisite genomic and proteomic databases for non-model organisms has enabled higher throughput characterization guided by predictive approaches. In silico tools such as Cysmotif Searcher [10] and SPADA [11] can be used to identify AMPs within these databases based on similarities with known peptide families. Then, mass spectrometric approaches can be used to detect predicted AMPs within peptide extracts and provide direct evidence for their translation [12]. Together, these tools facilitate the rapid identification of AMP-rich medicinal plant extracts for further characterization (e.g., activity characterization, mechanism of action studies, or structural elucidation).

Like other defense natural products, some plant Cys-rich AMPs are constitutively expressed, while others are only induced by environmental stress conditions or expressed in a specific tissue. For example, *Solanum tuberosum* snakin, StSN1, is constitutively expressed, while StSN2 is induced by wounding [13]. Additionally, *Viola hederacea* demonstrates tissue-specific expression of cyclotides, most significantly, expression differences between aerial tissue and tissues which are in contact with soil (e.g., roots, runners, and bulbs) [14]. Knowing the potential of tissue-specific AMP expression, seeds are an intriguing source of AMPs because they require chemical defenses at the ready to protect against a microbe-rich soil environment as they germinate [15,16].

Herein, we investigated the seeds of three edible plants used in traditional medicine: *Linum usitatissimum* (flax), *Trifolium pratense* (red clover), and *Sesamum indicum* (sesame). A variety of flax products (e.g., whole flaxseed, flaxseed oil, flaxseed meal, and flaxseed flour) are widely regarded as healthy dietary options [17]. Further, there is evidence that flaxseed can exhibit anticancer activity and improve blood lipid profiles [17]. In traditional medicine, red clover has been used in the treatment of heart disease, polycystic ovarian syndrome, bronchitis, fever, and cough [18]. Additionally, the antimicrobial and antifungal properties of red clover extracts were found to be active against *Staphylococcus aureus*, *Bacillus cereus*, *Escherichia coli*, *Aspergillus niger*, and *Candida albicans* [19]. Sesame, whose seeds are commonly consumed whole or used to produce cooking oil, is known to have an array of health benefits, including antibacterial, antifungal, antioxidant, and antitumor properties [20,21]. Sesame oil is known to demonstrate antibacterial activity against the common skin pathogens *Staphylococcus* and *Streptococcus* [22].

Though they are commonly consumed and used in ethnobotanical remedies, little is known about the Cys-rich AMPs produced by each species. Previous work in flax has focused on small, cysteine-free, cyclic peptides called orbitides, but largely ignored Cys-rich AMPs [23]. Sesame is known to produce an α-hairpinin as well as a 5.8 kDa unsequenced antibacterial peptide [24,25]. No peptide-level evidence of AMPs has been reported for red clover. This study combined in silico Cys-rich AMP prediction workflows to predict 80–100 Cys-rich AMPs from each species. Subsequent bottom-up proteomics analysis provided evidence for translation of 3–10 Cys-rich AMPs within the seeds of each species, including lipid transfer proteins, defensins, α-hairpinins, snakins, and plant albumins. Ten Cys-rich AMPs were identified within the sesame seed peptide extract, while nine and three were found in red clover and flax extracts, respectively. A preliminary antimicrobial bioactivity screen revealed no activity against a common *E. coli* lab strain, but additional testing against more robust panels including Gram-positive and Gram-negative bacteria could illuminate the antimicrobial breadth of these seed extracts. These results highlight the utility of a multi-pronged approach for the rapid prioritization of medicinal plant extracts, revealing that sesame and red clover seeds are strong targets for bioactive peptide discovery.

## 2. Results and Discussion

### 2.1. AMP Predictions

**Figure 2:**
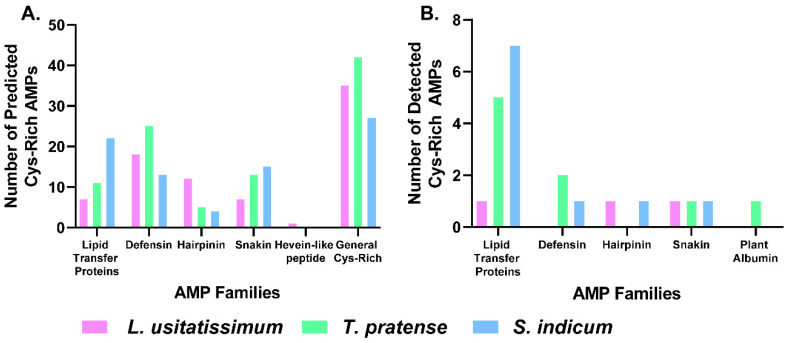
(**A**) Cys-rich AMP families predicted from the proteomes of *L. usitatissimum*, *T. pratense*, and *S. indicum*. (**B**) Cys-rich AMPs detected within seed peptide extracts.

SignalP and Cysmotif Searcher algorithms [10,26] were used in concert to predict 80, 96, and 81 AMPs within the proteomes produced from the sequenced genomes of *L. usitatissimum* [27], *T. pratense* [28], and *S. indicum* [29], respectively (Supplementary Table S1). The prediction of 80–100 AMPs is congruent with a recent analysis of 1267 plant transcriptomes revealing approximately 50–150 AMPs per plant species [30]. All three species included predictions of α-hairpinins, defensins, lipid transfer proteins, and unclassified Cys-rich peptides, with *L. usitatissimum* also containing a single hevein-like peptide (Figure 2A). Unclassified Cys-rich peptides were the most common category predicted in each species. These peptides contain a recognized Cys-motif embedded within a larger pattern of Cys residues which is not recognized. This can occur when analyzing precursor proteins with multiple AMP domains (e.g., hevein-like peptides and α-hairpinins) and other regions (e.g., linker or C-terminal pro-domains) which include additional Cys residue(s). Prediction of AMPs from genomes represents the pool of peptides that could be detected from *L. usitatissimum* [27], *T. pratense* [28], and *S. indicum* [29] samples; however, as plants are known to differentially express these peptides based on environmental and developmental conditions [13,14], we expected to only identify a subset within the medicinal plant seed extracts.

### 2.2. Proteomic Profiling of Seed Extracts

Traditional bottom-up proteomics was used to identify peptides present within *L. usitatissimum*, *T. pratense*, and *S. indicum* seed extracts (Supplementary Table S2). Among the identified peptides were 22 AMPs spanning 5 families (Table 1, Figure 2B). The predicted sequences of five unclassified Cys-rich peptides were compared to reviewed plant proteins deposited within Uniprot, revealing sequence similarity with α-hairpinins, snakins, and plant albumin 1bs, allowing more accurate classification of other detected Cys-rich AMPs (Supplementary Table S3).

Ten predicted AMPs were detected from the seeds of *S. indicum*, including seven lipid transfer proteins, two unclassified Cys-rich peptides, and one defensin. Similarly, lipid transfer proteins were the most common family of *T. pratense* identified and predicted AMPs, which included five lipid transfer proteins, two defensins, and two unclassified Cys-rich peptides detected in the peptide extract. *L. usitatissimum* yielded only three AMP identifications including a lipid transfer protein, an unclassified Cys-rich peptide, and a snakin. Overall, lipid transfer proteins were the most frequently detected peptide family despite not being the most common family within the predicted peptides. This is likely due to the generally high abundance of lipid transfer proteins in plant tissue [31], making them more amenable to detection than lower abundance peptides.

### 2.3. Lipid Transfer Proteins

**Figure 3:**
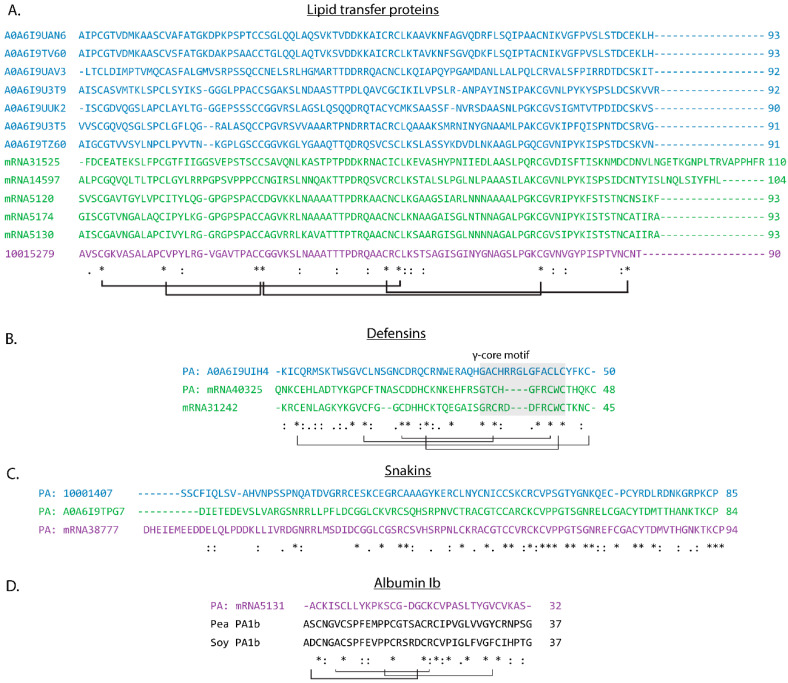
Sequence alignment of Cys-rich AMPs detected in *S. indicum* (blue), *T. pratense* (green), and *L. usitatissimum* (purple). (**A**) Lipid transfer proteins identified in seed extractions across all plant extracts share a common Cys-motif. (**B**) Defensins from *S. indicum* and *T. pratense* contain the expected Cys motif forming four disulfide bonds and the characteristic γ-core motif (grey). (**C**) Snakins identified in seed extract tryptic digests. (**D**) Sequence alignment of plant albumin 1b peptides identified in flax, pea (Uniprot: P62926), and soybean (Uniprot: Q39837). Asterisks note fully conserved residues, two dots note positions with highly similar residues, and single dots note positions with weakly similar residues.

Lipid transfer proteins (LTPs) from *L. usitatissimum*, *T. pratense*, and *S. indicum* have been primarily studied via transcriptomics rather than as mature peptides [32,33,34,35]. LTPs accounted for 13 of 22 Cys-rich AMPs identified across the three seed extracts. LTPs are relatively high-molecular-weight AMPs (7–9 kDa) containing four disulfide bonds which stabilize four or five α-helices [36]. They are considered pathogenesis-related proteins and possess a variety of biological activities, including antibacterial, antifungal, antiviral, and enzyme activity inhibitory properties [37]. Lipid transfer proteins also contribute to important biological functions such as membrane stabilization, reproduction, and development [38]. LTPs are classified in two categories (LTP1 and LTP2) based on features such as disulfide bonding patterns (I–VI, II–III, IV–VII, and V–VIII vs. I–V, II–III, IV–VII, and VI–VIII, respectively), approximate peptide lengths (90–95 vs. 65–70 amino acids, respectively), and secondary structure (4 α-helices vs. 3 α-helices, respectively) [37]. Each LTP identified within *L. usitatissimum*, *T. pratense*, and *S. indicum* seed extracts is at least 90 residues in length, with sequence alignment which revealed conserved Cys residues that are consistent with the LTP1 subfamily (Figure 3A).

### 2.4. Defensins

Defensins are broadly distributed across the kingdoms of life and are among the most well-studied plant AMP families, with more than 1200 known members [39]. In plants, they typically contain four disulfide bonds and are approximately 50 residues in length [39]. They are best recognized for their activity against agriculturally relevant fungal pathogens, although other activities (e.g., antibacterial, α-amylase inhibitory, and anticancer properties) are receiving increased attention [40,41,42]. Some defensins have additional roles in functions such as heavy metal tolerance and root growth [43,44]. Previous reports have identified changes in *T. pratense* defensin transcript abundance in response to stress but did not provide evidence of peptide accumulation [45]. Three predicted defensins in total were identified within the *T. pratense* and *S. indicum* seed extracts, representing the first peptide-level evidence for the accumulation of defensins in *T. pratense* and *S. indicum* seed extracts.

Sequence alignment confirmed the presence of eight Cys residues expected to form four disulfide bonds (I–VIII, II–V, III–VI, and IV–VII) which is consistent with structurally characterized plant defensins (Figure 3B) [39]. Each detected defensin also contained the defensin γ-core motif (GXCX_3-9_C, where X_n_ is the number of residues between cysteines) (Figure 3B) [46]. This region is important to the antibacterial and antifungal activity of plant defensins and has been used to generate truncated synthetic analogs of mature defensins [47]. γ-Core motif-containing synthetic peptides are much shorter than mature defensins (~1.2 kDa vs. 6 kDa) and do not include disulfide bonds, which makes them more tractable synthetic targets [5,47,48,49,50,51,52]. Antimicrobial assays assessing the activity of truncated synthetic analogs of the *T. pratense* and *S. indicum* defensins described here could be used for the rapid prioritization of mature defensins for sequence characterization, isolation, and mechanism of action studies.

### 2.5. α-Hairpinins

More than 20 α-hairpinins have been discovered in a variety of species including grasses, squashes, and maize but are generally less thoroughly characterized than defensins or lipid transfer proteins [25,53]. Members of this lesser-known Cys-rich AMP family have demonstrated antibacterial, antifungal, and trypsin inhibitory activities [53]. They contain two disulfide bonds that crosslink two antiparallel α-helices [53]. N-terminal conversion of glutamic acid or glutamine to pyroglutamic acid is commonly observed in this family of peptides and can provide additional resistance against proteolysis [53]. α-Hairpinins are often translated as part of larger precursor proteins which can contain multiple α-hairpinin domains and may be buried within functional proteins [25,53]. Although some α-hairpinins appear to be excised by asparaginyl endopeptidase (AEP)-mediated cleavage (hydrolysis C-terminal to D and N), this does not appear to be a universal trend within the family [53,54]. As a result, this family is a challenge for plant AMP-predictive workflows which remain unable to identify the additional proteolysis events needed for α-hairpinin maturation or to predict which of the multiple α-hairpinin domains are processed into mature peptides. Vicilins, a family of seed storage proteins, are known functional proteins that can be proteolytically cleaved to yield α-hairpinins, leading to the alternative name “vicilin-buried peptides” [25].

**Figure 4:**
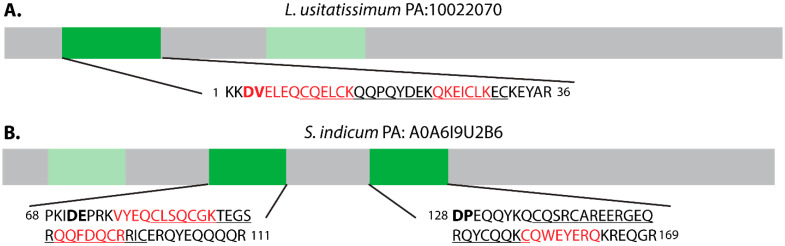
Organization of α-hairpinin-containing proteins predicted from (**A**) *L. usitatissimum* precursor accession (PA): 10022070 and (**B**) *S. indicum* PA: A0A619U2B6. α-Hairpinin domains are shaded dark green if detected in tryptic seed extract and light green if not detected. Sequences including each detected α-hairpinin motifs (underlined) and surrounding residues are shown. Tryptic peptides detected in seed extract are red. Bold text indicates potential AEP cleavage sites.

Two predicted AMPs containing α-hairpinin motifs were identified in Linum *usitatissimum* and *Sesamum indicum* seed extracts, and each was originally categorized as an unclassified Cys-rich peptide prior to re-classification due to sequence similarity with vicilins (Supplemental Table S3). The α-hairpinin identified in *L. usitatissimum* (Precursor accession (PA): 10022070) contains two α-hairpinin domains, but tryptic peptides were only identified from the first α-hairpinin domain (Figure 4A). Similarly, tryptic peptides were detected from two of the three α-hairpinin domains identified within an *S. indicum* predicted peptide (PA: A0A6I9U2B6) (Figure 4B), again suggesting that not all α-hairpinin domains within the same precursor accumulate as mature peptides. This trend is further supported by the previous characterization of VBP-9, a vicilin-buried peptide from *S. indicum* seeds, which also was the only mature product detected within a peptide extract of a vicilin precursor containing multiple α-hairpinin domains [25]. 

The sequences surrounding each of the α-hairpinin motifs detected here contain potential N-terminal AEP cleavage sites (Figure 4, bold). However, tryptic peptide coverage of the *L. usitatissimum* α-hairpinin suggests that cleavage does not occur at the AEP site, and tryptic peptides encompassing the N-terminal AEP cleavage sites of *S. indicum* α-hairpinins were not detected. VBP-9, previously detected in *S. indicum* seeds, appears to be processed by AEP, providing precedence for the role of this protease in sesame α-hairpinin maturation [25]. However, unlike VBP-9, neither sesame α-hairpinin detected here appears to have C-terminal AEP processing sites.

### 2.6. Snakins

The snakin/GASA family are ~6 kDa peptides with six disulfide bonds translated within a precursor containing an N-terminal signal peptide, leader peptides, and a snakin/GASA domain [55]. Although more than 10 snakin-like peptides have been confirmed, thousands more have been predicted in genomes and transcriptomes, but their disulfide bonding pattern has only been experimentally confirmed once [7,55]. Snakins are generally considered antimicrobial, while GASA peptides are related to growth and development; however, the families are difficult to differentiate without peptide activity assays [55]. A total of three putative snakins (PA: 10001407, PA: A0A6I9TPG7, and PA: mRNA38777) were identified from *L. usitatissimum*, *T. pratense*, and *S. indicum* seed extracts (Table 1). Two of the putative snakins (PA: A0A6I9TPG7 and PA: A0A6I9TPG7) were re-classified from uncharacterized Cys-rich peptides to snakins based on sequence comparison with known snakins (Supplemental Table S3). Sequence alignment highlighted the conserved snakin Cys motif (Figure 3C). Recent research has suggested that disulfide formation may not be essential for snakin antimicrobial activity, indicating that a synthetic linear analog may be feasible for future bioactivity screening [56,57].

### 2.7. Plant Albumins

Plant albumins 1b (PA1b) are 4 kDa insecticidal peptides derived from albumin and contain three disulfide bonds that form a knottin motif [58,59]. They are translated within albumin-1 proteins which contain Cys residues within other peptide domains [58], causing PA1b precursors to elude proper classification via Cysmotif Searcher. Comparison of the predicted Cys-rich AMP from *T. pratense* (PA: mRNA5131) revealed sequence similarity with albumin-1 proteins from *Glycine soja* (wild soybean), *Pisum sativum* (pea), and *Glycine max* (soybean) (Supplemental Table S3), including conserved Cys residues within known PA1b domains (Figure 3D). Furthermore, all tryptic peptides identified were within the PA1b domain, supporting the hypothesis that the mature AMP was present within the sample. This is the first evidence for the accumulation of a PA1b AMP in *T. pratense*.

### 2.8. Bioactivity Assessment

Despite proteomic evidence supporting the presence of multiple AMPs per species, flax, red clover, and sesame seed fractions did not demonstrate antibacterial activity against *Escherichia coli* (Supplemental Figure S1). This demonstrates a limitation of utilizing solely a bioactivity-guided approach for AMP discovery. A dynamic range of peptide concentrations, varying MIC ranges, and activity specificity limit the discovery of AMPs in an extract within a singular bioassay. Further, plant Cys-rich AMPs are increasingly recognized as multi-functional, not only exhibiting antimicrobial activity but also contributing to functions such as growth and development, heavy metal tolerance, and abiotic stress resistance [38,43,44,55]. As a result, Cys-rich AMP-like peptides extracted from plant material may have alternative primary biological functions. A prediction-guided discovery platform yields higher throughput identifications of translated AMPs, and subsequent screening of these peptide-rich extracts or synthetic peptides derived from them against a more robust panel has the potential to reveal novel activities and enrich the general understanding of plant Cys-rich AMP biological function.

## 3. Conclusions

*L. usitatissimum*, *T. pratense,* and *S. indicum* are edible traditional medicinal plants whose cysteine-rich AMPs are underexplored. This study combined peptide predictions with bottom-up proteomics to profile AMPs from the seeds of each species. In silico predictions revealed 80–100 putative AMPs within the genome of each species. These predictions represent the total pool of predicted peptides that possess the cysteine motifs characteristic of antimicrobial peptide families and could be expressed by these plants. However, plants are known to differentially express AMPs based on tissue type, growth conditions, environment, etc. [13,14]. As a result, coupling in silico predictions with bottom-up proteomic screening of plant extracts facilitates the rapid prioritization of botanical samples for AMP discovery based on peptide expression. Furthermore, these data justify further bioactivity probing, including expanding to a more robust panel against Gram-positive and Gram-negative bacteria, when the initial antimicrobial screen against *E. coli* was unsuccessful. Here, bottom-up proteomics was used to identify 22 novel peptides spanning five AMP families and revealed that the higher number of Cys-rich AMPs detected in sesame and red clover seeds make these plants a higher priority for future investigation than flax, despite inactivity of all fractions against *E. coli.* Furthermore, this work lays the foundation for future targeted peptidomics studies to characterize the mature intact AMPs and define proper proteolytic processing, post-translational modifications, and bioactivity. This work, which may include isolation from extracts or synthesis of prioritized AMPs, will further our understanding of the contributions of botanical AMPs within diet and traditional medicines.

## 4. Materials and Methods

### 4.1. AMP Prediction

Protein databases derived from the genomes sequences of *Linum usitatissimum* (Phytozome, v1.0, accessed 15 May 2021) [27], *Trifolium pratense* (Phytozome, v2, accessed 15 May 2021) [28], and *Sesamum indicum* (Uniprot, UP000504604, accessed 15 May 2021) [29] were submitted to SignalP-5.0 [26] to identify proteins that contained signal peptides and predict cleavage sites. A FASTA file with these proteins after signal peptide cleavage was exported and submitted to Cysmotif Searcher (version 3.31) [10] to predict AMPs within each proteome. With Cysmotif Searcher, the option of skipping translation of input sequences was used, and SPADA [11] was not included in the computational pipeline.

### 4.2. Peptide Extraction and Fractionation

*Linum usitatissimum*, *Trifolium pratense,* and *Sesamum indicum* seeds were purchased from Strictly Medicinal Seeds (Williams, OR, USA). Peptide extracts were created as previously described with modifications noted here [60]. Briefly, pulverized seeds were extracted in an acetic acid solution (5 g seeds/150 mL acid solution) with size-exclusion steps to remove large proteins (>30 kDa) and small molecules (<1 kDa) and fractionated using strong cation-exchange chromatography (SCX) to remove neutral and negatively charged molecules. The crude extract was concentrated to 3 mL, and 450 µL injections were fractionated on a PolySulfethyl A column (100 mm × 4.6 mm, 3 µm particles, PolyLC, Columbia, MD, USA) with a 30 min linear gradient of mobile phase A (5 mM ammonium formate, 20% acetonitrile, pH 2.7) to mobile phase B (500 mM ammonium formate, 20% acetonitrile, pH 3.0) with a 0.5 mL/minute flow rate, collecting a single fraction from 10 to 30 min per species. SCX eluates were concentrated, desalted, and fractionated using a Sep-Pak C_18_ column (100 mg, Waters, Milford, MA, USA), sequentially eluting in 80/20/0.1 water/acetonitrile/formic acid, 60/40/0.1 water/acetonitrile/formic acid, 40/60/0.1 water/acetonitrile/formic acid, and 20/80/0.1 water/acetonitrile/formic acid. Sep-Pak eluates were collected, producing a total of four fractions per species and concentrated to dryness in a vacuum centrifuge, eliminating volatile formic acid prior to resuspension in 50 μL of LC-MS-grade water for further analysis.

### 4.3. Reduction, Alkylation, and Trypsin Digestion

Reduced (dithiothreitol) and alkylated (iodoacetamide) or reduced, alkylated, and trypsin-digested peptide fractions were prepared for LC-MS/MS, as previously described [12]. All samples were desalted using C_18_ Ziptips (Millipore Sigma, Burlington, MA, USA) prior to LC-MS analysis.

### 4.4. LC-MS/MS Data Acquisition

LC-MS/MS data for bottom-up proteomic analysis of *L. usitatissimum*, *T. pratense,* and *S. indicum* seed fractions were acquired using an Acquity M-class UPLC system (Waters, Milford, MA, USA) coupled to a Q Exactive HF-X Hybrid Quadrupole-Orbitrap mass spectrometer (Thermo Scientific, Waltham, MA, USA) as previously described [61]. Briefly, mobile phase A consisted of water with 0.1% formic acid (Thermo Scientific, Waltham, MA, USA), and mobile phase B was acetonitrile with 0.1% formic acid (Thermo Fisher Scientific, Waltham, MA, USA). Injections were made into a Symmetry C_18_ trap column (100 Å, 5 μm, 180 μm × 20 mm; Waters, Milford, MA, USA) with a flow rate of 5 μL/min for 3 min using 99% A and 1% B. Peptides were then separated on an HSS T3 C_18_ column (100 Å, 1.8 μm, 75 μm × 250 mm; Waters, Milford, MA, USA) using a linear gradient of increasing mobile phase B at a flow rate of 300 nl/min. Mobile phase B was held at 5% for 1 min, then increased from 5% to 50% in 30 min before ramping to 85% in 2 min, where it was held for 3 min before returning to 5% in 1 min and re-equilibrating for 23 min. The mass spectrometer was operated in positive polarity, and the Nanospray Flex source had spray voltage floating at 2.1 kV, capillary temperature at 320 °C, and funnel RF level at 40. MS survey scans were collected with a scan range of 350–2000 m/z at a resolving power of 120,000 and an AGC target of 3 × 10^6^ with a maximum injection time of 50 ms. A top 20 data-dependent acquisition was used where HCD fragmentation of precursor ions having +2 to +7 charge state was performed using a normalized collision energy setting of 28. MS/MS scans were performed at a resolving power of 30,000 and an AGC target of 1 × 105 with a maximum injection time of 100 ms. Dynamic exclusion for precursor *m*/*z* was set to a 10 s window. Collected raw MS data (*.raw) were converted to Mascot Generic Files (*.mgf) using ProteoWizard MSconvert [62].

### 4.5. Database Searching and Peptide Identification

Database searching was performed against one of the following species-specific protein databases appended with the sequences for common laboratory contaminants (www.thegpm.org/cRAP; 116 entries): *Linum usitatissimum* (Phytozome, v1.0, accessed 15 May 2021, 43,484 entries) [27], *Trifolium pratense* (Phytozome, v2, accessed 15 May 2021, 41,297 entries) [28], and *Sesamum indicum* (Uniprot, UP000504604, accessed 15 May 2021, 21,374 entries) [29]. Digests were searched (Matrix Science, version 2.5.1) against the appropriate database and a decoy database using peptide/fragment mass tolerances 15 ppm/0.02 Da, trypsin specificity, three possible missed cleavages, and a fixed modification of cysteine carbamidomethylation. Peptide false discovery rates (FDR) were adjusted to ≤1% using the Mascot Percolator algorithm [63]. Peptides with a Mascot score >13, matching to *Linum usitatissimum*/*Trifolium pratense*/*Sesamum indicum* entries, and at least one unique tryptic peptide were considered for further analysis. The identified proteins were manually parsed for predicted AMPs. The mass spectrometry proteomics data have been deposited to the ProteomeXchange Consortium (http://proteomecentral.proteomexchange.org) via the PRIDE partner repository [64] with the data set identifier PXD029289.

### 4.6. Sequence Alignment

The sequences of Cysmotif Searcher predicted peptides that were identified in *L. usitatissimum*, *T. pratense*, and *S. indicum* seed fractions as classified as Cys-rich were compared to those in the known Uniprot database of reviewed plant proteins using the Basic Local Alignment Search Tool (BLAST) to identify any similarities with known AMP families [65]. Peptide sequence alignment figures were prepared with ClustalOmega [66].

### 4.7. Bioactivity Assay

Bioassay of *L. usitatissimum*, *T. pratense,* and *S. indicum* seed stepped-elution fractions were performed in triplicate in a 96-well plate format against *Escherichia coli* ATCC 25922. Peptide fractions concentrations were normalized based on the mass of seed extracted (0.15 g seeds/10 µL water). Bacterial cultures were inoculated in 5 mL of Mueller Hinton Broth (MHB) and grown for 16 h at 37 °C with shaking (250 rpm) to an optical density at 600 nm (OD_600_) of 0.25. After incubating for an additional hour, the cultures were added to the 96-well plate already containing 10 µL of fraction or control along with 10 µL of 2× MHB and 1× MHB for a final volume of 50 µL and OD_600_ of 0.1 per well. Ampicillin (0.1 mg/mL) and LC-MS-grade water were used as positive and negative controls, respectively. The 96-well plate was incubated for 4 h at 37 °C with shaking (250 rpm) before recording the OD_600_ of each well. Percent activity was calculated as shown below with Equation (1):

(1) $$Percent\;activity=\left(1-\frac{OD_{600}\left(sample\right)-\;OD_{600}\left(positive\;control\right)}{OD_{600}\left(negative\;control\right)-OD_{600}\left(positive\;control\right)}\right)*100$$

## Figures and Tables

**Table 1:** Cys-rich AMPs identified in *Sesamum indicum* (sesame), *Trifolium pratense* (red clover), and *Linum usitatissimum* (flax) seed peptide extract tryptic digests. Sequences of tryptic peptides identified in Mascot database search are red. Asterisks (*) indicate AMPs that were recategorized from the Cysmotif Searcher “Cys-rich” category based on sequence similarity with other AMPs.

Plant Species	Precursor Accession	Family	Predicted Sequences	Mascot Score
*S. indicum*	A0A6I9UIH4	Defensin	KICQRMSKTWSGVCLNSGNCDRQCRNWERAQHGACHRRGLGFACLCYFKC	26
*S. indicum*	A0A6I9UUK2	Lipid transfer protein	ISCGDVQGSLAPCLAYLTGGGEPSSSCCGGVRSLAGSLQSQQDRQTACYCMKSAASSFNVRSDAASNLPGKCGVSIGMTVTPDIDCSKVS	1373
*S. indicum*	A0A6I9U3T5	Lipid transfer protein	VVSCGQVQSGLSPCLGFLQGRALASQCCPGVRSVVAAARTPNDRRTACRCLQAAAKSMRNINYGNAAMLPAKCGVKIPFQISPNTDCSRVG	591
*S. indicum*	A0A6I9TV60	Lipid transfer protein	AIPCGTVDMKAASCVSFATGKDAKPSAACCTGLQQLAQTVKSVDDKKAICRCLKTAVKNFSGVQDKFLSQIPTACNIKVGFPVSLSTDCEKLH	275
*S. indicum*	A0A6I9UAN6	Lipid transfer protein	AIPCGTVDMKAASCVAFATGKDPKPSPTCCSGLQQLAQSVKTVDDKKAICRCLKAAVKNFAGVQDRFLSQIPAACNIKVGFPVSLSTDCEKLH	146
*S. indicum*	A0A6I9TZ60	Lipid transfer protein	AIGCGTVVSYLNPCLPYVTNKGPLGSCCGGVKGLYGAAQTTQDRQSVCSCLKSLASSYKDVDLNKAAGLPGQCGVNIPYKISPSTDCSKVN	35
*S. indicum*	A0A6I9U3T9	Lipid transfer protein	AISCASVMTKLSPCLSYIKSGGGLPPACCSGAKSLNDAASTTPDLQAVCGCIKILVPSLRANPAYINSIPAKCGVNLPYKYSPSLDCSKVVR	26
*S. indicum*	A0A6I9UAV3	Lipid transfer protein	LTCLDIMPTVMQCASFALGMVSRPSSQCCNELSRLHGMARTTDDRRQACNCLKQIAPQYPGAMDANLLALPQLCRVALSFPIRRDTDCSKIT	14
*S. indicum*	A0A6I9TPG7	Snakin*	DIETEDEVSLVARGSNRRLLPFLDCGGLCKVRCSQHSRPNVCTRACGTCCARCKCVPPGTSGNRELCGACYTDMTTHANKTKCP	18
*S. indicum*	A0A6I9U2B6	α-hairpinin*	YTNPQLQEGEEESAEEGLFKCFVSCEKRRENEHELSQCEKRCVREYQERKREEREERGGRRGEETVVPKIDEPRKVYEQCLSQCGKTEGSRQQFDQCRRICERQYEQQQQREKRGGGEGTIENHHRRDPEQQYKQCQSRCAREERGEQRQYCQQKCQWEYERQKREQGREQGGGGGSTNPRKEREEEEEQEGKNPYFFESQRFDSKYRTEEGNVKVLERFSKKSELLQGVDNYRLAVLEANPNTFVLPHHFDAESVLVVAGGKGTISYVWQNRRKSYNVKLGDVMRVPAGSIVYLVNRDDNEKLYVLKLLQPVNTPGRFKEYFGVGGENPESFYRTFSNEILEAAFNVPSDRLKRLFGQQKKGVIIRASKEQIRALSQESEESSRGRREESWGPFNLLEGRPLFSNRYGQYFEASPNDYQQLKDLDVSVGFMNINKGGMVAPYYNSRSTKLVLVVGGNGRFEMACPHRSARSKQGRKERQGETTDVRYQRVSARLSIGDAFIVPAGHPIAMIASQDSNLQLVSFGIKGSYNQKYFLAGQDNIWNQVESEAKELSFKMPAREVEEIFRRQEQSYFLPGPGQGEERGKEHYVASILDFVGF	315
*T. pratense*	mRNA40325	Defensin	QNKCEHLADTYKGPCFTNASCDDHCKNKEHFRSGTCHGFRCWCTHQKC	119
*T. pratense*	mRNA31242	Defensin	KRCENLAGKYKGVCFGGCDHHCKTQEGAISGRCRDDFRCWCTKNC	27
*T. pratense*	mRNA31525	Lipid transfer protein	FDCEATEKSLFPCGTFIIGGSVEPSTSCCSAVQNLKASTPTPDDKRNACICLKEVASHYPNIIEDLAASLPQRCGVDISFTISKNMDCDNVLNGETKGNPLTRVAPPHFR	3106
*T. pratense*	mRNA5174	Lipid transfer protein	GISCGTVNGALAQCIPYLKGGPGPSPACCAGVKRLNAAAATTPDRQAACNCLKNAAGAISGLNTNNAGALPGKCGVNIPYKISTSTNCATIRA	520
*T. pratense*	mRNA5130	Lipid transfer protein	AISCGAVNGALAPCIVYLRGGRGPSPACCAGVRRLKAVATTTPTRQAACNCLKSAARGISGLNNNNAGALPGRCGVSIPYKISTSTNCAIIRA	451
*T. pratense*	mRNA14597	Lipid transfer protein	ALPCGQVQLTLTPCLGYLRRPGPSVPPPCCNGIRSLNNQAKTTPDRQSVCRCLKSTALSLPGLNLPAAASILAKCGVNLPYKISPSIDCNTYISLNQLSIYFHL	128
*T. pratense*	mRNA5120	Lipid transfer protein	SVSCGAVTGYLVPCITYLQGGPGPSPACCDGVKKLNAAAATTPDRKAACNCLKGAAGSIARLNNNAAAALPGKCGVRIPYKFSTSTNCNSIKF	29
*T. pratense*	mRNA5131	Plant albumin 1b*	ACKISCLLYKPKSCGDGCKCVPASLTYGVCVKASFEHVTNMVEEHPNLCESHDDCTKKGSGSFCARFPNPEIEYGWCFDSNSHAQASFKNAQESSNFFLKMPSAIST	96
*T. pratense*	mRNA38777	Snakin*	DHEIEMEEDDELQLPDDKLLIVRDGNRRLMSDIDCGGLCGSRCSVHSRPNLCKRACGTCCVRCKCVPPGTSGNREFCGACYTDMVTHGNKTKCP	22
*L. usitatissimum*	10015279	Lipid transfer protein	AVSCGKVASALAPCVPYLRGVGAVTPACCGGVKSLNAAATTTPDRQAACRCLKSTSAGISGINYGNAGSLPGKCGVNVGYPISPTVNCNT	205
*L. usitatissimum*	10001407	Snakin	SSCFIQLSVAHVNPSSPNQATDVGRRCESKCEGRCAAAGYKERCLNYCNICCSKCRCVPSGTYGNKQECPCYRDLRDNKGRPKCP	63
*L. usitatissimum*	10022070	α-Hairpinin*	KKDVELEQCQELCKQQPQYDEKQKEICLKECKEYARKKSGRGSEETDPEKRLEECKHQCKQHKFSDEEQKKACRTKCDKQYKEGRGRIGTYYYYEEEQEESKGENPYVFTEEHFESKSQSQHGRVDVLRKFTDKSELLKGIENFRIGFLEANPQTFVPPAHFDADGVFFVAQGRGTFTMIEGNRGRMTSSSEIKRHSFNIEAGDVVRVYAGSPVYLVNKHESQKLVIIKFIRPVNLPGSFDAFHGPGGENPESFFRAFSPELLAAAFKVDKQRIQRIFQQQEGEILKATREQIRALSHGEEGGGIWPFGGESTGPFNLLHRRPTQKNTFGQLWEADPNEFEQFRDLDLLVSFANITQGAMAGPFYNSKATKIAYVVNGEGYFEMACPHVTSSSGDMGRQTRGSQSRGGQKYGKVRSQLRRGTVFIVPAGHPVVTVASANNNLEVLCFEVNAQGNFRFSLAGKDNVMSKMESEALELGFGAPAREVEQIFKNRNEEFFFPGPEWQKQQHSRGYSSA	453

## Data Availability

Publicly available datasets were analyzed in this study. These data can be found here: ProteomeXchange Consortium (http://proteomecentral.proteomexchange.org) via the PRIDE partner repository with the data set identifier PXD029289 (Reviewer account details: Username: reviewer_pxd029289@ebi.ac.uk, Password: cnkoh1TN).

## Supplementary Materials

The following are available online. Supplementary_spreadsheet.xls contains Table S1: Cys-rich AMPs predicted from the proteomes of *S. indicum*, *T. pratense*, and *L. usitatissimum*, Table S2: Significant Mascot identifications from *L. usitatissimum*, *T. pratense*, and *S. indicum* seed fraction, and Table S3: Similarity of Cysmotif Search-predicted general Cys-rich peptides identified in seed extracts with proteins from the Uniprot database of plant proteins. Supplemental_figures.pdf contains Figure S1: Bioactivity of *S. indicum*, *T. pratense*, and *L. usitatissimum* seed extract fractions against *E. coli*.

## Sample Availability

Medicinal plants seed samples of the compounds are available from the authors upon request.
