# Supplementary material for: Mass Spectrometric Identification of Antimicrobial Peptides from Medicinal Seeds
Source: Molecules. 2021 Dec 1;26(23):7304. doi: 10.3390/molecules26237304 (PMC8659199; doi:10.3390/molecules26237304)
Supplement: Supplementary file 1 [file molecules-26-07304-s001.zip › Supplementary material/Supplemental_figures.pdf]

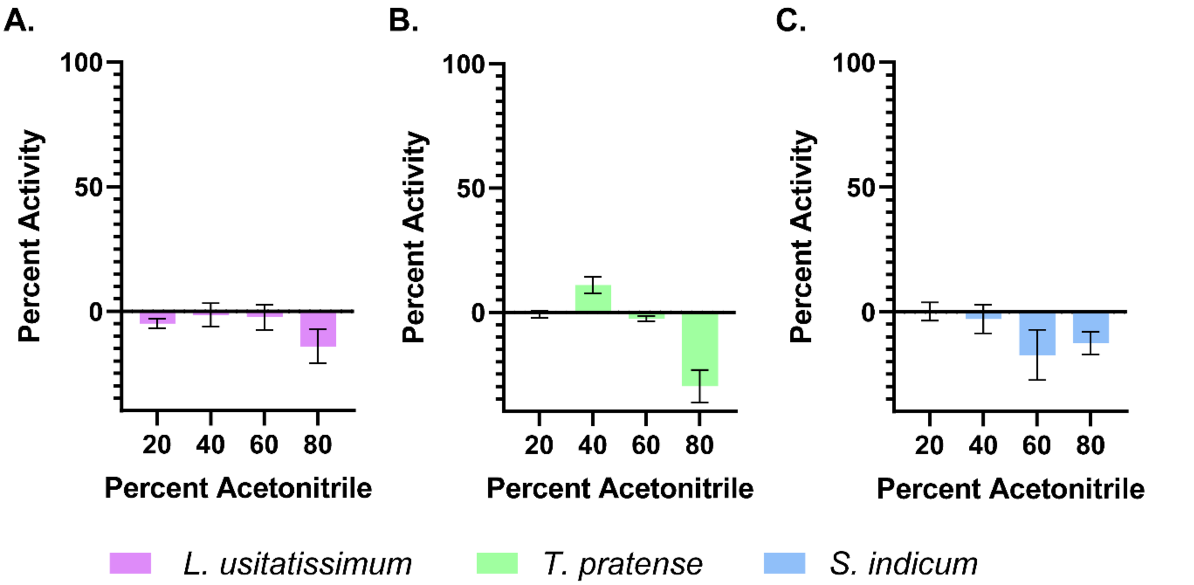

Supplemental Figure S1. Bioactivity assays of *L. usitatissimum* (A), *T. pratense* (B), and *S. indicum* (C) seed extract fractions sequentially eluted in 20, 40, 60, and 80% acetonitrile screened against *E. coli* ATCC 25922. Error bars are shown as ± standard deviations of three replicates.
